# Bone Diseases of the Jaws

**DOI:** 10.1155/2010/702314

**Published:** 2010-03-31

**Authors:** Pieter Johannes Slootweg

**Affiliations:** Department of Pathology, Radboud University Nijmegen Medical Center, HP 824, P. O. Box 9101, 6500 HB Nijmegen, The Netherlands

## Abstract

Lesions specific for the jaws and not occurring in any other bones mostly are related to the teeth or to odontogenic tissues. Moreover, the jaws may harbor nonodontogenic bone lesions not seen in any other part of the skeleton. This paper pays attention to the diseases that are specific for the jaws, odontogenic as well as nonodontogenic. Both neoplastic and nonneoplastic entities will be discussed.

## 1. Introduction

Jaw bone differs from other bones in several aspects. Embryologically, it is unique due to its development from cells migrating from the embryonal neuroectoderm. Anatomically it houses the tooth germs. With both unique features in the jaws, diseases occur that are not seen in any other part of the skeleton. For the purpose of this paper, these will be divided in 2 main groups: those related to the dentition and those restricted to the bone proper. Tooth-related jaw bone diseases can be divided in cysts and odontogenic tumors. Reactive bone diseases, fibro-osseous lesions, giant cell lesions, and bone tumors are taken together as the main second group.

## 2. Cysts of the Jaws

The majority of cysts in the jaw bones are called odontogenic as they are derived from odontogenic epithelium that is related with the tooth development; only a few are nonodontogenic but have other kinds of epithelium as their source. Odontogenic cysts are classified as inflammatory and developmental. In the first group, inflammatory changes are the stimulus for epithelial odontogenic remnants to proliferate and transform into epithelium-lined cystic cavities. This means that their occurrence can be related to poor oral health. Appropriate dental care will reduce their incidence. The pathogenesis of the developmental cysts is not elucidated [1, 2].

### 2.1. Odontogenic Cysts: Inflammatory

*Radicular cysts* are located at the root tips of teeth with necrotic pulp tissue, mostly due to extensive caries. Histologically, they are lined by nonkeratinizing epithelium. The fibrous cyst wall may show a chronic inflammatory infiltrate composed of lymphocytes and plasma cells. The extent of this infiltrate may vary. A radicular cyst left behind in the jaw after removal of the associated tooth is called *residual cyst. *Cysts with the same histology as mentioned above but located at the lateral side of the tooth at the border between enamel and root cementum are called *paradental.* It is thought that they may be related to deep periodontal pockets.

### 2.2. Odontogenic Cysts: Developmental

*Dentigerous cysts* surround the crown of an unerupted tooth, mostly the maxillary canine or the mandibular third molar tooth. Usually, the cyst wall is attached at the neck of the involved tooth, forms an umbrella covering the crown part, and is lined by an epithelial lining consisting of two to three layers of cuboidal cells. These cysts may be big in size and radiologically, they can be confused with odontogenic tumors. In practice, the larger the pericoronal radiolucency, the higher the chance that it is not a dentigerous cyst but a genuine neoplastic odontogenic lesion. *Lateral periodontal cysts* are located between the roots of vital teeth. This discerns them from laterally positioned radicular cysts which are related to nonvital teeth. They are lined by a thin, nonkeratinizing squamous or cuboidal epithelium with focal, plaque-like thickenings that consist of clear cells. Sometimes, cysts with this histological appearance occur in the soft tissues of the gingiva: then the designation *gingival cyst* is employed.* Botryoid odontogenic cysts *also have the same histology but are much larger and multilocular by radiology.

The *glandular odontogenic cyst*, also called *sialo-odontogenic cyst* shows an epithelial lining that is partly nonkeratinizing squamous, partly cuboidal or columnar with cells that can have cilia and may form papillary projections. It is important to recognize this cyst histologically as it may recur after treatment.

**Figure 1:**
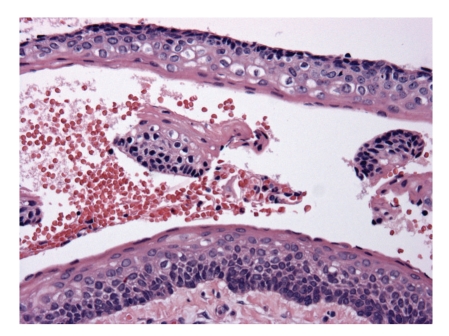
Picture of keratocyst showing typical epithelial lining with basal palissading and corrugating parakeratinized surface.

From a practical point of view, the only cyst for which classification goes beyond academic value is the* odontogenic keratocyst*. Histologically, this cyst differs from all other jaw cysts in showing a lining of stratified squamous epithelium with a well-defined basal layer of palissading columnar or cuboidal cells and a superficial corrugated parakeratin layer (Figure 1). The underlying cyst wall may contain tiny daughter cysts and solid epithelial nests; they are more common in cysts associated with the nevoid basal cell carcinoma syndrome [3]. Recognition is important as keratocysts tend to recur after enucleation and sometimes, a partial jaw resection is needed for cure. Because of this neoplastic behavior, the most recent WHO classification proposes the diagnostic designation *keratocystic odontogenic tumor* for this lesion [4]. Although this reclassification is based on clinical features, its rationale is supported by the demonstration of genetic alterations that are common for neoplasia such as loss of tumor suppressor gene activity and overexpression and amplification of other genes [5]. Sometimes, cysts are lined by orthokeratinized epithelium, thus having the appearance of an epidermoid cyst. They should be distinguished from the odontogenic keratocyst with parakeratinization because they lack any tendency for recurrence, thus being amenable to more limited treatment than the odontogenic keratocyst with the parakeratinizing epithelium.

### 2.3. Nonodontogenic Cysts

As the name already implies, these cysts are derived from other epithelial sources in the jaws or neighbouring soft tissues.

Epithelial remnants of the nasopalatine duct are the source for the *nasopalatine duct cyst. *The cyst lies in the anterior palate just behind the central incisor teeth; its lining consists of an admixture of squamous and respiratory epithelium. If one of both neighbouring central incisor teeth are nonvital, differentiation between a nasopalatine duct cyst and a radicular cyst may become impossible.

The nasolacrimal duct may give origin to the* nasolabial cysts.* This cyst is located in the soft tissue just lateral to the nose at the buccal aspect of the maxillary alveolar process. Its lining also combines squamous and respiratory elements. Due to its soft tissue location, there are no associated radiologic abnormalities.

## 3. Odontogenic Tumors

Odontogenic tumors comprise a group of lesions that have in common that they arise from the odontogenic tissues. As tooth germs have both an epithelial and a mesenchymal part, these tumors may be either epithelial, mesenchymal, or both.

Clinically, this group of lesions encompasses entities whose behavior varies from frankly neoplastic including metastatic potential to nonneoplastic hamartomatous. The latter may recapitulate normal tooth development including the formation of dental hard tissues such as enamel, dentin, and cementum [6]. It is obvious that a precise diagnosis is mandatory; at one hand to avoid unnecessary overtreatment in case of hamartomatous lesions with their limited growth potential and subsequent maturation, at the other hand to avoid delay in treatment of genuine odontogenic neoplasms. Due to overlapping histological features, this is not always easy.

### 3.1. Odontogenic Tumors: Epithelial

*Ameloblastomas* are among the more common odontogenic tumors. They consist of epithelial strands or of discrete epithelial islands. The cells at the border with the adjacent fibrous stroma are columnar and resemble ameloblasts, the cells that form enamel in the immature tooth. Liquefaction in the epithelial as well as in the stromal areas may cause cysts that coalesce to form the large cavities responsible for the multicystic gross appearance ameloblastomas may show. The tumor infiltrates the adjacent cancellous bone. Therefore, treatment should include removal of some adjacent healthy jaw bone to obtain tumor-free margins [7].

From the various known subtypes only the desmoplastic and the unicystic warrant further discussion. *Desmoplastic ameloblastoma* shows a dense collagenous stroma, the epithelial component being reduced to narrow strands of epithelium and within the stromal component, and active bone formation can be observed [8]. This type of ameloblastoma may be confused with a bone forming jaw lesion, especially one of the fibro-osseous group, as the bone formation in the stroma leads to a mixed radiodense-radiolucent radiological appearance. This contrasts with the radiological appearance of prototypical ameloblastomas that are homogeneously radiolucent.

**Figure 2:**
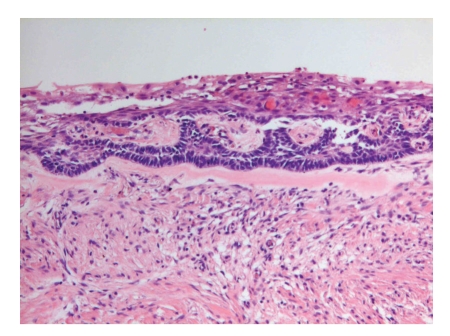
Unicystic ameloblastoma. Epithelial lining shows basal rim of dark staining palissading cells and subepithelial hyalinization which both are typical for ameloblastoma.

Sometimes, ameloblastomas present themselves as cysts consisting of one single intraosseous cavity that is lined by ameloblastomatous epithelium (Figure 2). This type is called *unicystic ameloblastoma. *If there is “dropping off” of this epithelium into the underlying fibrous cyst wall, the behavior is the same as that of the conventional ameloblastoma thus necessitating adequate surgery. The uncomplicated unicystic ameloblastoma without this mural epithelial component may be treated by simple enucleation [9]. To avoid overlooking an intramural component, extensive histological sampling of the enucleated cyst wall is required.

Less common is the *calcifying epithelial odontogenic tumor, *a lesion composed of sheets of large eosinophilic cells with pleomorphic nuclei and very conspicuous intercellular bridges. Growth characteristics are the same as for the ameloblastoma and hence, treatment also includes removal with a healthy margin [10].

**Figure 3:**
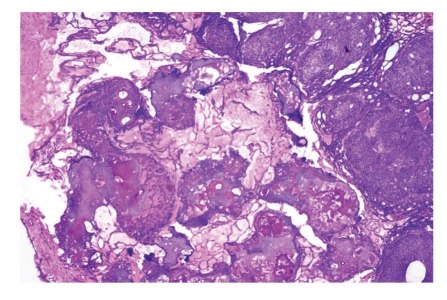
Adenomatoid odontogenic tumor consisting of epithelial nodules containing calcified material and interconnected with thin epithelial strands.

*Adenomatoid odontogenic tumor *quite often assumes the clinical presentation of a dentigerous cyst, investing the crown part of an impacted tooth to which it is connected at the level of the cemento-enamel junction. Histologically, this tumor consists of epithelial nodules connected to each other by thin epithelial strands. Larger nodules also contain duct-like spaces lined by columnar cells and extracellular eosinophilic matrix (Figure 3). Its behavior is benign and simple enucleation is adequate treatment.

The last entity in the group of epithelial odontogenic tumors is the s*quamous odontogenic tumor. *It is composed of islands of well-differentiated nonkeratinizing squamous epithelium surrounded by mature fibrous connective tissue. In the epithelial islands, cystic degeneration as well as calcification may occur. Invasion into cancellous bone may be present.

### 3.2. Odontogenic Tumors: Mesenchymal

*Odontogenic myxomas* consist of monotonous cells with a fibroblastic appearance that lie in a myxoid stroma. This histomorphology is almost identical to the dental follicle and the embryonic dental pulp, to so-called dental papilla. To avoid misdiagnosing these components of the normal tooth germ as myxoma, clinical and radiographic data are decisive [11]. As the tumor does not show encapsulation, it has to be removed including a rim of adjacent normal jaw bone to avoid recurrence [12]. So, it will be apparent that proper distinction between an immature dental pulp and myxoma is mandatory to avoid unnecessary mutilating surgery at one side or delaying the treatment needed for myxoma at the other side.

*Odontogenic fibroma* is a controversial entity. Uncertainty exists about the histologic spectrum that this lesion may show as well as about its separation from other fibrous jaw lesions. The lesion consists of fibroblasts lying in a background of myxoid material intermingled with collagen fibers that may vary from delicate to coarse and thus resembling the dental follicle [11, 13].

*Cementoblastomas* consist of a mass of cellular cementum connected with the root surface that may show signs of external resorption. In addition to the hard tissue-component, fibrous tissue with hyperplastic cementoblasts is present [14]. Cementoblastomas should not be confused with hypercementosis, an increase in thickness of the cemental layer that covers the root surface and that may be both cellular and acellular. Rarely, confusion may arise concerning the distinction of cementoblastoma from osteoblastoma; the latter is a bone tumor more common to the extragnathic skeleton. As osteoblastomas lack the firm connection with the roots of the teeth, radiographic documentation allows the differentiation between both [15]. Clinically, cementoblastomas are characterized by persistent pain which is rather unique for benign bone forming jaw lesions.

### 3.3. Odontogenic Tumors: Mixed Epithelial and Mesenchymal

Mixed odontogenic tumors contain both epithelial and mesenchymal tissues and may recapitulate odontogenesis in varying degrees [16].

**Figure 4:**
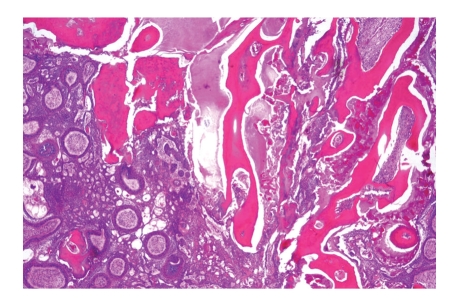
Ameloblastic fibro-odontoma showing ameloblastomatous epithelium, embryonal myxoid pulp tissue and dentin as well as enamel matrix.

When the lesion resembles the immature tooth germ without presence of either dentin or enamel, it is called *ameloblastic fibroma.* If there is a combination of dentin as well as enamel together with soft tissues resembling the epithelial enamel organ and the mesenchymal dental papilla, the lesion is diagnosed as an *ameloblastic fibro-odontoma *(Figure 4). In case of a rather prominent epithelial component, the ameloblastic fibroma may be confused with ameloblastoma. As the ameloblastic fibroma can be treated with simple enucleation whereas ameloblastomas require major resection, distinction between both is very important. This distinction is based on the stromal connective tissue component, immature myxoid in ameloblastic fibroma and fibrous, and mature in ameloblastoma. Radiologically, no reliable distinguishing features are present. As ameloblastomas do not contain dental hard tissue, distinction from ameloblastic fibro-odontoma does not pose major difficulties. Radiographs show a radiolucent lesion in case of ameloblastoma and a mixed radiodense-radiolucent lesion in case of ameloblastic fibro-odontoma.

If there are no immature odontogenic tissues present, lesions entirely consisting of an admixture of dentin and enamel, they are called *odontomas *[16]. In* complex odontoma,* the dental hard tissues show an haphazard arrangement; in c*ompound odontoma* they form tiny teeth. In a single lesion, the number of these teeth may vary from a few to dozens.

Some of the mixed odontogenic tumors mimic ameloblastoma by a predominance of an epithelial component similar to this tumor. However, they can be distinguished from ameloblastoma because of the concomitant presence of large, pale epithelial cells without a well-defined nucleus, the so-called ghost cells, and the presence of dentin. These lesions are called* calcifying cystic odontogenic tumor * when they contain a central cystic cavity and *dentinogenic ghost cell tumor *if they manifest themselves as a solid tumor mass [17, 18]. In the past, both entities were taken together under the common term calcifying odontogenic cyst or Gorlin cyst. Their distinction from ameloblastoma is important as they can be treated conservatively.

### 3.4. Odontogenic Tumors: Malignant

Both odontogenic epithelium as well as odontogenic mesenchyme may show neoplastic degeneration, causing either odontogenic carcinomas or odontogenic sarcomas [19]. As they all are very rare, they will be discussed very briefly, only mentioning the most important features. Within the group of carcinomas, one discerns the following entities: *malignant (metastasizing) ameloblastoma* is an ameloblastoma that metastasizes in spite of an innocuous histologic appearance. The primary tumor shows no specific features different from ameloblastomas that do not metastasize. *Ameloblastic carcinoma* is characterized by cells that, although mimicking the architectural pattern of ameloblastoma, exhibits an histomorphology indicating malignancy with the corresponding behavior: invasive growth and metastasis. The resemblance to ameloblastoma distinguishes this tumor from a *primary intraosseous carcinoma* which is a well to poorly differentiated squamous cell carcinoma arising within the jaw; not derived from the oral mucosa but probably from odontogenic epithelial remnants or an odontogenic cyst. 

Odontogenic sarcomas are characterized by pleomorphic fibroblastic cells. Depending on whether they contain only soft tissues or also dentin or both dentin and enamel, they are called *ameloblastic fibrosarcoma, ameloblastic fibrodentinosarcoma, *or *ameloblastic fibro-odontosarcoma*.

## 4. Reactive Bone Lesions

**Figure 5:**
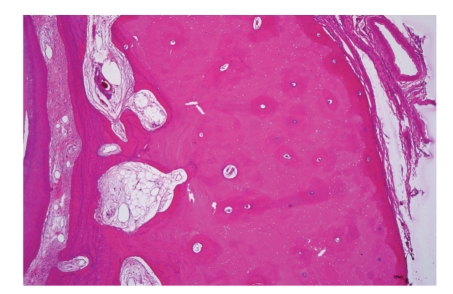
Alveolar tooth socket thickened due to formation of torus. The torus consists entirely of lamellar bone. At the left side the periodontal ligament space with the tooth surface are shown.

Reactive bone lesions occur in 2 different forms. The first is the so-called *tori.* These lesions are bony outgrowths of the cortical bone (Figure 5). Mostly, they are found in the palatal midline, buccally at the maxillary alveolar ridge or both buccally and lingually at the mandibular alveolar ridge. They may hamper the applicability of dental prostheses. Otherwise, they can be left untouched, not requiring any treatment.

**Figure 6:**
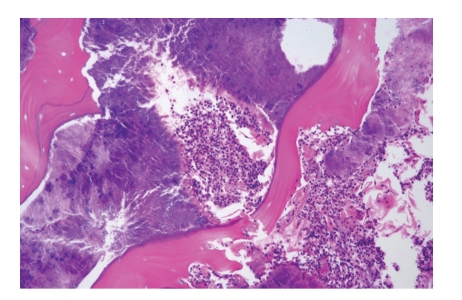
Acute osteomyelitis. Bony sequestrae are surrounded by colonies of bacteria as well as purulent infiltrate.

**Figure 7:**
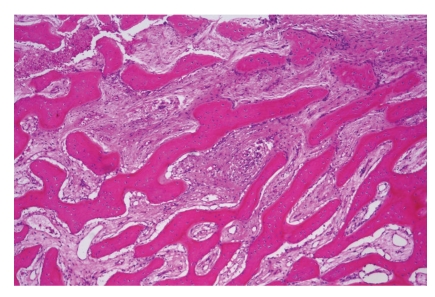
Chronic osteomyelitis. Parallel arrangement of lamellar bone trabeculae and intervening edematous marrow with sparse lymphocytes are typical for this disease.

The second group to be mentioned under this heading is the inflammatory diseases. The presence of diseased teeth may represent a porte d'entrée for micro-organisms causing infection of the jaw bone. If bone necrosis and pus formation are predominant, one speaks of acute osteomyelitis (Figure 6). Unless the oral surgeon removes the dead bone, the disease will not heal. In case of low-grade infection, fibrosis and bone sclerosis are observed (Figure 7). This chronic osteomyelitis has to be differentiated from fibrous dysplasia or osseous dysplasia (see below for distinguishing features).

### 4.1. Fibro-Osseous Lesions

**Figure 8:**
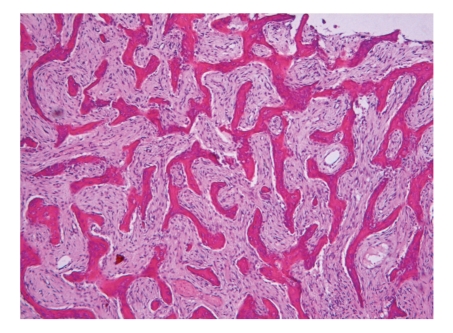
Fibrous dysplasia. Irregular trabeculae of woven bone lie in a monotonous fibrous stroma.

Fibro-osseous lesions are characterized by the presence of bone marrow that has changed into fibrous tissue and that contains mineralized material of varying appearances. Depending on the proportions of soft and mineralized tissue, they may be predominantly radiolucent, mixed radiodense-radiolucent, or mainly radiodense. Because of overlapping clinical, radiological, and histopathological features, their classification has evoked much discussion that probably will continue. Their current classification recognizes fibrous dysplasia, ossifying fibroma, and osseous dysplasia [20]. *Fibrous dysplasia* occurs in three clinical subtypes: monostotic which affects one bone, polyostotic which affects multiple bones, and Albright's syndrome in which multiple bone lesions are accompanied by skin hyperpigmentation and endocrine disturbances. Histologically it is composed of cellular fibrous tissue containing trabeculae of woven bone (Figure 8). Activating missense mutations of the gene encoding the *α* subunit of the stimulatory G protein are a consistent finding in the various forms of fibrous dysplasia. This genetic feature of fibrous dysplasia probably will be the convincing argument against the opinion that fibrous dysplasia and ossifying fibroma (to be discussed next) are merely opposite ends of a clinical and radiological spectrum that encompasses one single entity [21]. 

*Ossifying fibroma*, formerly also called *cemento-ossifying fibroma*, is composed of fibrous tissue that contains woven as well as lamellar bone and acellular mineralized material resembling cementum. Its circumscribed nature and variation in cellularity and types of mineralized tissues distinguishes ossifying fibroma from fibrous dysplasia as does the absence of the specific genetic alteration occurring in the latter and mentioned above.

**Figure 9:**
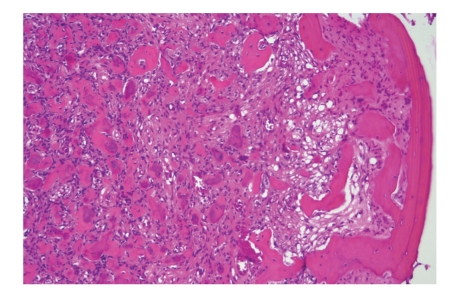
Ossifying fibroma, juvenile psammomatoid variant. Irregular ossicles lie in fibrous stroma. At the right side, an expanded cortical bone layer is present.

Recently identified subtypes of ossifying fibroma are *juvenile trabecular* and *juvenile psammomatoid ossifying fibroma*. The former shows bands of cellular osteoid together with slender trabeculae of plexiform bone lined by a dense rim of enlarged osteoblasts. This lesion may be confused with osteosarcoma. Its favoured site is the upper jaw. The latter is characterized by small ossicles resembling psammoma bodies, hence its name (Figure 9). This type usually is located in the walls of the sinonasal cavities but sometimes can be encountered in the mandible [22].

**Figure 10:**
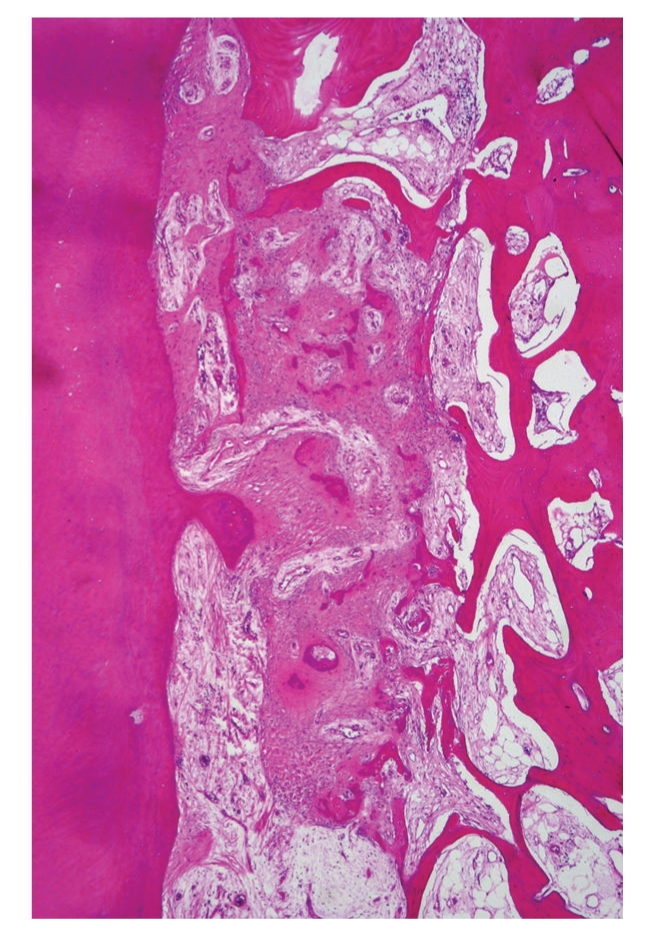
Osseous dysplasia. Between root surface (left) and alveolar socket (right) the periodontal ligament shows the presence of irregular bony particles.

*Osseous dysplasia* occurs in 3 different clinical forms. *Periapical osseous dysplasia* occurs in the anterior mandible and involves only a few adjacent teeth. A similar limited lesion occurring in a posterior jaw quadrant is known as *focal osseous dysplasia. Florid osseous dysplasia* is larger, involving 2 or more jaw quadrants and *familial gigantiform cementoma* involves multiple quadrants while being expansile. This latter type of osseous dysplasia shows an autosomal dominant inheritance. All 3 subtypes have the same histomorphology: cellular fibrous tissue, trabeculae of woven as well as lamellar bone, and spherules of cementum-like material (Figure 10) [23]. The immature form of periapical osseous dysplasia radiologically shows a periapical radiolucency. This should not be interpreted as periapical disease necessitating endodontic treatment. Vitality tests will be of diagnostic value in making the right decision, nonvitality in case of periapical disease and vitality in case of periapical osseous dysplasia. This point especially concerns the mandibular frontal teeth.

### 4.2. Giant Cell Lesions

**Figure 11:**
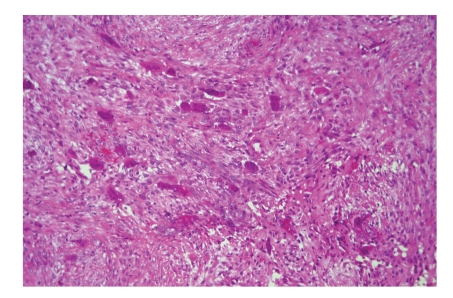
Cherubism: osteoclast-like giant cells lie in a fibroblastic background. Central giant cell granuloma shows an identical picture; therefore distinction between both is made on clinical and radiological grounds.

*Central giant cell granuloma* and *cherubism* both show osteoclast-like giant cells lying in a fibroblastic background tissue that may vary in cellularity from very dense to cell-poor (Figure 11). The giant cells mostly cluster in areas of haemorrhage but they also may lie more dispersed among the lesion. Giant cell granuloma and cherubism are distinguished by the younger age of occurrence and the involvement of two or more jaw quadrants by the latter. Moreover cherubism has a genetic etiology, the responsible alteration having been localized to chromosome 4p16.3 [24].

The expansion of the affected jaw areas causes the angelic face leading to the lesion's designation: cherubism. With the onset of puberty, the lesions loose their activity and may mature to fibrous tissue and bone. Treatment of cherubism consists of cosmetic recontouring the maxillofacial bones if needed for cosmetic reasons. For persistent or recurrent giant cell granuloma there are some indications that treatment with calcitonin may be beneficial [25].

## 5. Conclusions

This overview of lesions in the jaw bones illustrates the huge variety occurring at this site. The clinician who is responsible for diagnosis and treatment of patients with jaw swellings should realize that quite often, there is considerable overlap in both clinical, histological, as well as radiological features. Diagnostic errors can have big consequences by causing inappropriate therapy, either too extensive or too limited. Moreover, dental and periodontal infections may both mimic or hide more serious afflictions of the jaw. Therefore a good assessment of dental and periodontal status is the first step in evaluation of patients with any jaw disease.
